# The Melanoma Antigens MELOE-1 and MELOE-2 Are Translated from a *Bona Fide* Polycistronic mRNA Containing Functional IRES Sequences

**DOI:** 10.1371/journal.pone.0075233

**Published:** 2013-09-25

**Authors:** Delphine Carbonnelle, Virginie Vignard, Delphine Sehedic, Agnes Moreau-Aubry, Laetitia Florenceau, Maud Charpentier, Wolfgang Mikulits, Nathalie Labarriere, François Lang

**Affiliations:** 1 Institut National de la Santé et de la Recherche Médicale, U892, Nantes, France; 2 University of Nantes, Nantes, France; 3 Centre national de la recherche scientifique, UMR 6299, Nantes, France; 4 CHU Nantes, Nantes, France; 5 Institute of Cancer Research, Comprehensive Cancer Center, Vienna, Austria; University of Tennessee, United States of America

## Abstract

Our previous studies on melanoma antigens identified two new polypeptides, named MELOE-1 and MELOE-2, that are involved in immunosurveillance. Intriguingly, these antigens are coded by distinct open reading frames (ORF) of the *meloe* mRNA which is significantly expressed only in the melanocytic lineage. In addition, MELOE-1 and -2 specific T cell clones recognized melanoma cells but very poorly normal melanocytes suggesting differential translation of *meloe* in normal *vs* tumor cells. This prompted us to elucidate the mechanisms of translation of these antigens in melanoma cells. We first demonstrated that no splicing event or cryptic promoter could generate shorter *meloe* transcripts containing only one of the two ORFs. Triggering *meloe* RNA degradation with a siRNA close to the ORF coding for MELOE-2 abrogated expression of both MELOE-1 and MELOE-2, thus confirming that the two ORFs are always associated. Next we showed, in a bicistronic reporter system, that IRES activities could be detected upstream of MELOE-1 and MELOE-2 and finally confirmed their translation from full length *meloe* cDNA in melanoma cells with eGFP constructs. In conclusion, *meloe* is a polycistronic mRNA that generates both MELOE-1 and MELOE-2 antigens through IRES-dependent translation in melanoma cells and that may explain their tumor specificity.

## Introduction

Several clinical trials have suggested that T cell immunotherapy may be worthwhile in cancer and particularly in metastatic melanoma [1]. In this respect, tumor antigens that can be recognized by T lymphocytes have been classified into mutated, differentiation, tumor-specific and over-expressed antigens [2]. Regardless of this classification, most of the tumor antigens and especially melanoma antigens are translated from the main large open reading frame (ORF) of their mRNA by a classical translation pathway i.e. cap-dependent ribosome scanning and translation at an initiation codon within a Kozak sequence. However, alternative translation mechanisms that can generate neo antigens and cryptic T cell epitopes have been described [3]. The most frequent occurrence is the initiation of translation from an alternative start codon located within the main ORF of a previously identified antigen. In melanoma, this was reported within ORFs coding for gp75 [4], NY-ESO-1 [5], LAGE-1 [6] and BING-4 [7]. In addition, translation of a short ORF from a processed pseudogene (i.e. in which the main ORF is invalidated) can lead to T cell epitope generation in melanoma as we previously reported [8]. More recently, it was reported in prostate cancer and chronic myeloid leukemia, that a short ORF located downstream of the main ORF coding for myotrophin was translated by an IRES (Internal Ribosomal Entry Sequence)-dependent mechanism to generate the novel MPD6 antigen [9].

Our group has been working for many years on the characterization of melanoma antigens relevant for adoptive T cell therapy. In the course of our studies, we identified a novel melanoma antigen, named MELOE-1, that was recognized by tumor-infiltrating lymphocytes (TIL) infused to patients who remained relapse-free after adoptive cell transfer in an adjuvant setting [10]. Surprisingly, MELOE-1 was translated from a small ORF of the *meloe* mRNA that contained only multiple short ORFs. Later on, we demonstrated that another ORF from the same mRNA could be translated into a second antigen, coined MELOE-2, that also triggered T cell responses in melanoma patients [11]. This raised several questions concerning the mechanisms of MELOE-1 and 2 production: is *meloe* a true polycistronic mRNA or do shorter transcripts of *meloe* mRNA exist in melanoma cells that could allow independent translation of each ORF? If *meloe* is polycistronic, what is the mechanism of ORF translation?

Analysis of the transcriptomes in eukaryotes revealed a significant number of polyA containing transcripts lacking long ORFs [12], [13] and were thus considered as non coding RNA. However, more recent studies, notably in insects, have provided evidence that a mRNA with short ORFs could be translated into physiologically relevant peptides [14], [15], [16], [17], [18].

In the present work, we used various approaches to demonstrate that *meloe* mRNA is truly polycistronic and that the translation of at least two of its ORFs in melanomas (MELOE-1 and 2) is dependent on IRES sequences.

## Materials and Methods

### Cell Lines and T Cell Cultures

Melanoma cell lines established from fragments of metastatic tumors are registered in the Biocollection PC-U892-NL (CHU Nantes). The colon carcinoma cell line, SW480, was purchased from ATCC (CCL228). All cell lines were maintained in RPMI 1640 containing 10% fetal calf serum (FCS) (Sigma, Lyon, France). The two CD8+ T cell clones specific for MELOE-1 and MELOE-2 [10], [19] were grown in RPMI 1640 8% HS supplemented with 150 IU/ml of IL-2 (Chiron, France).

### Plasmid Constructions

**Figure 1:**
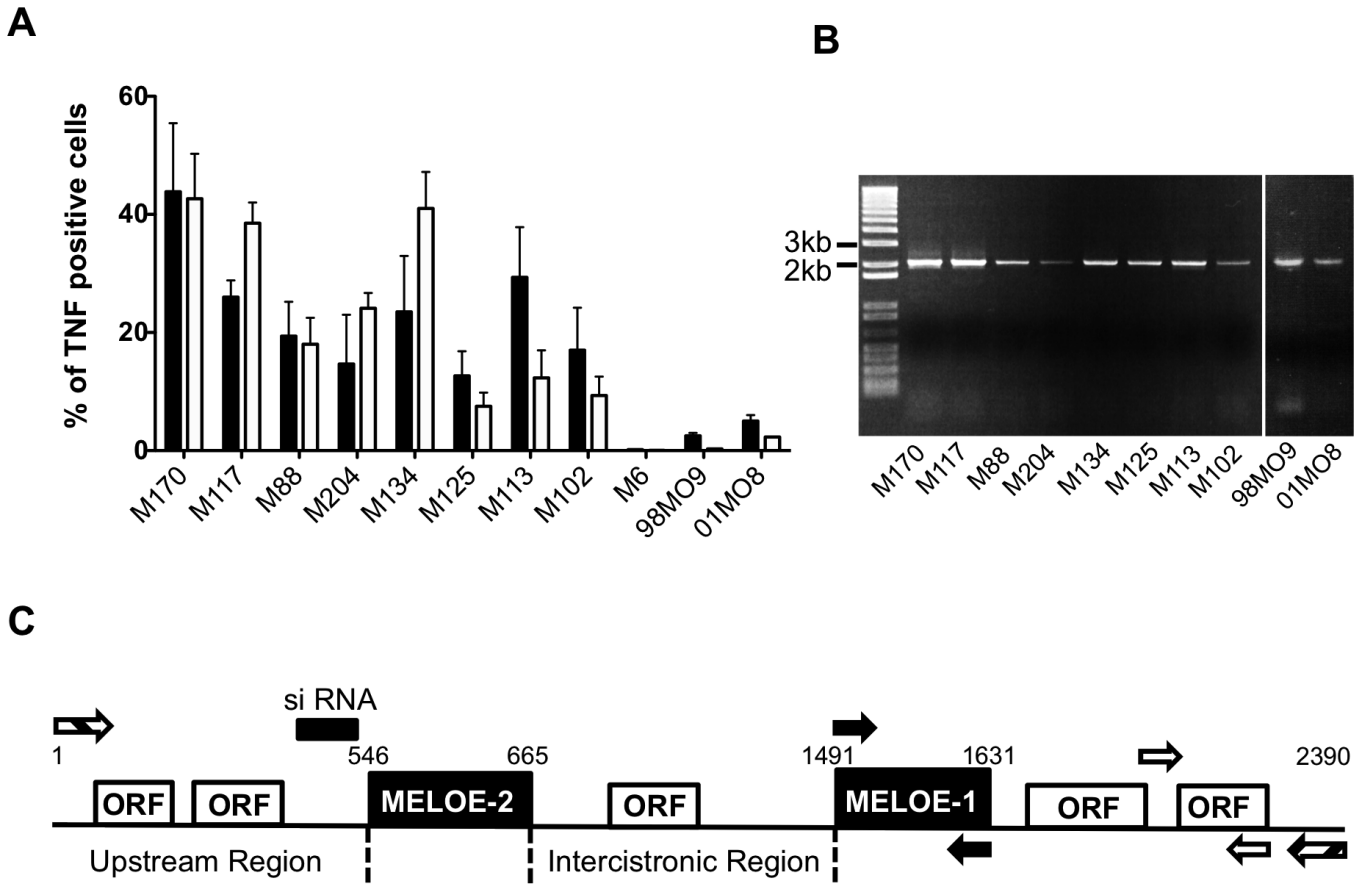
Expression of MELOE-1 and MELOE-2 antigens and *meloe* mRNA in the melanocytic lineage. A. TNF secretion by MELOE-1 (black bars) and MELOE-2 (white bars) T cell clones in response to HLA-A2 positive melanoma cells, melanocytes (98MO9, 01MO8) or the HLA-A2 negative M6 cell line. Cells were co-cultured at a 1∶2 clone:cell ratio and TNF-α responses were assessed by intracellular staining. Bars represent mean ±SEM of three independent experiments. B. Full length RT-PCR products amplified from cell lines RNA samples with *meloe* 5′ and 3′primers. C. Structure of *meloe* cDNA. Boxes represent ORFs. Arrows indicate primer sets: *meloe* 5′-3′endpoint PCR (hatched) or qPCR in the MELOE1 region (black) or in the 3′ downstream region (white). The siRNA, Upstream and Intercistronic regions are illustrated.

For transfection into melanoma and SW480 cells, full length *meloe* cDNA intact or modified with enhanced Green Fluorescent Protein (eGFP) cDNA (made by GenArt, Life Technologies, St-Aubin, France) was cloned into a pcDNA3 expression vector (Invitrogen, Life Technologies). Bicistronic expression vectors containing Renilla and Firefly luciferase ORFs (pRF) with or without the IRES from EMCV virus (pR-EMCV-F) were previously described (Petz *et al.,* 2007). To look for IRES activity, cDNA sequences located between ORF_546–665_ and ORF_1491–1631_ or before the ORF_546–665_ (figure 1c) were cloned into pRF. For cryptic promoter assays, the SV40 promoter of the PGL4 vector (Promega, Charbonnières, France) was replaced by the Intercistronic Region (IR) or MELOE-2 Upstream Region (UR) (figure1C). The 600 pb melanA promoter [20] was used as positive control.

### Transient Transfection and Reporter Analyses

Melanoma or colon carcinoma cells at 50–70% confluency were transfected with plasmids (4 µg/10^6^ cells) and LTX lipofectamine (Invitrogen) according to the manufacturer’s instructions. For luciferase reporter assays, cells were lysed 48 h post-transfection and luminescence was measured with a VICTOR X3 (Perkin Elmer, Courtaboeuf, France) and expressed in arbitrary units. eGFP fluorescence was examined 48 h post transfection with an automated fluorescence High Content Screening (HCS) microscopic system (Array Scan VTI, ThermoScientific, Courtaboeuf, France). Nuclear staining was performed with 20 µM Hoeschst 33342 (Sigma). Overlay fluorescent images of Hoechst-stained nuclei and GFP labelled cells were acquired using 386/420 nm and 485/515 nm excitation/emission filters with a 10X objective. Forty-nine fields per well were imaged resulting in the counting of over 8000 cells per condition (Cellomics® View software, Thermo Fisher Scientific).

### RT-PCR Analysis

Total RNA was extracted from melanoma or colon carcinoma cell lines after transfection with NucleoSpin RNAII kit (Macherey-Nagel, Hoerdt, France). Prior to reverse transcription, DNA was removed from each sample with Turbo DNase (Ambion, Life Technologies). Retrotranscription was performed using 1 µg of total RNA, oligo dT, and Superscript III reverse transcriptase (Invitrogen). A negative control was included that contained no reverse transcriptase to check for absence of residual plasmid. All samples were amplified with the Phusion Hot Start Polymerase (Thermo Scientific) using *meloe* forward (5′-ATCCCCACCCACCCGGCTCC-3′) and reverse (5′-CATGACATGCCCGCATTTCC-3′) primers. PCR conditions were 98°C for 30 s, followed by 30 or 35 cycles of 98°C for 10 s, 70°C for 30 s, 72°C for 2 min, followed by additional 72°C for 10 min.

### SiRNA Tranfection

A siRNA (5′-GAGUUUCACGUGGCAGUCCGGUAAA-3′) targeting *meloe* mRNA was designed to hybridize 55 bp upstream of MELOE-2 ORF_546–665_ (Ambion). A universal control siRNA (Sigma) was used as a negative control. SiRNAs (50 nM) were transfected into melanoma cells using lipofectamine 2000 (Invitrogen) according to the manufacturer’s instructions. A reporter plasmid coding for eGFP protein (pEGFP-N3, Clontech, Ozyme, St-Quentin-en-Yvelines, France) was co-transfected with the siRNAs to select transfected cells. After 48 h, eGFP positive cells were sorted with a FACS ARIA III (BD Biosciences, Le-Pont-de-Claix, France) and used for qPCR or T cell stimulations.

### Real-time PCR Analysis

Total RNA was extracted with NucleoSpin RNAII kit and retrotranscription was performed as above. Relative quantification of *meloe*, and housekeeping genes *rplpo* and *cyclophilin-A* expression was performed using Brilliant SYBR Green QPCR (Stratagene, Agilent technologies, Les-Ulis, France). 20 ng of cDNA was added to 200 nM of SYBR green Mix in 25 µL. Primers were: 5′-ATCCCCACCCACCCGGCTCC-3′ and 5′-CATGACATGCCCGCATTTCC-3′ for MELOE-1 primers and 5′-GTCCTCCCCAGCACCAGAGT-3′ and 5′-AGCCTGCCAT CTGCAATCCT-3′ for 3′DR primers. *RPLPO* primers were: 5′-GTGATGT GCAGCTGATCAAGACT-3′ and 5′-GATGACCAGCCCAAAGGAGA-3′; and *cyclophilin-A* primers were: 5′-CCACCGTGTTCTTCGACAT-3′ and 5′-CCAGTGCTCAGAGCACGAAA-3. Thermal cycling was one step at 95°C for 10 min, followed by 40 cycles at 95°C for 30 s, 60°C for 1 min, and 72°C for 1 min. Mean threshold cycle (*CT*) values from duplicate PCR reactions were normalized to mean *CT* values for two housekeeping genes (cyclophilin-A and RPLPO) from the same cDNA preparations. The relative expression ratio of a target gene was calculated as follows considering the PCR efficiency (*E*) and the *CT* deviation between a given cell line (*x*) and a reference cell line (*calibrator*):
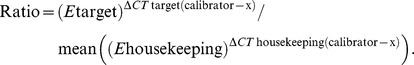



### Immunoprecipitation and Western Blot

Transfected cells were lysed with Tris pH 7,6 10 mM, NaCl 140 mM, EDTA 1 mM, Triton X100 1% with protease inhibitors. Protein content of lysates was quantified by BCA assay. Total lysates (1,5 mg) were denaturated by incubation with 2% SDS for 10 min at 70°C, diluted in PBS and precipitated overnight with mouse anti-eGFP mAb (Clontech, Ozyme, France) at 4°C. Protein G PLUS-Agarose (Santa Cruz Biotechnology, Heidelberg, Germany) was added (30 µl/tube), and the mixture was incubated under rotation for 2 h at 4°C. After washes, bound proteins were eluted by incubation for 10 minutes at 70°C in 30 µL of 2×Laemmli sample buffer. Whole IP samples and 5 µL of control eGFP-transfected cells were run on a 12% SDS-PAGE gel and blotted onto Immobilon®-P PVDF membranes (Millipore, Molsheim France). Membranes were probed with the same anti-eGFP mAb and HRP-conjugated secondary antibody followed by ECL detection (Bio-Rad, Marnes-la-Coquette, France). Membrane staining was analysed with ChemiDoc™ MP ImagingSystem apparatus (BioRad).

### Functional Analysis of T Cells

Lymphocytes were stimulated for 5 h in the presence of brefeldin A (10 µg/mL, Sigma) with tumor cell lines at an E:T ratio of 1∶2. Cells were then fixed with 4% paraformaldehyde (Sigma), permeabilised with saponine 0.1% and stained with an APC-conjugated anti-TNF-α specific antibody (Miltenyi Biotec, Paris, France) as previously described [10] and analyzed by flow cytometry.

### Statistical Analysis

Results are expressed as mean±SEM. All results were compared using ANOVA analysis and a Dunnett post-test.

## Results

### Expression of MELOE-1 and MELOE-2 in Melanoma and Melanocyte Cell Lines

Expression of MELOE-1 and MELOE-2 at the protein level was evaluated indirectly with two T cell clones, clone M170.48 [10] and clone M170.51 [11] that are specific for a HLA-A2-restricted MELOE-1 and MELOE-2 epitope, respectively. We tested the capacity of eight HLA-A0201+ melanoma cell lines and two HLA-A0201+ melanocyte cell lines to stimulate TNFα production by the two clones. As shown, each of the 8 melanoma cell lines stimulated both clones, with variable intensities in TNF responses while normal melanocytes were poorly stimulatory (figure 1A). The HLA-A2-negative melanoma cell line M6 was not stimulatory. These data therefore suggested the simultaneous expression of both antigens in all melanoma cell lines and a very low expression in normal melanocytes.

RT-PCR amplification using primers located at the 5′ end and 3′ end of the *meloe* transcript showed that melanocytes also expressed detectable levels of *meloe* (figure 1B). Considering that these two co-expressed antigens are coded by distinct ORFs within *meloe* mRNA (figure 1C), we also looked for the presence of alternate *meloe* transcripts that may contain only one of the two ORFs. As shown on figure 1B, in our PCR conditions, no other amplicon than the full length 2091 bp fragment could be detected neither in melanoma cell lines nor in melanocytes.

### Expression of MELOE-1 and MELOE-2 in the Colon Carcinoma SW480 Cell Line Transfected with the Full Length *meloe* cDNA

**Figure 2:**
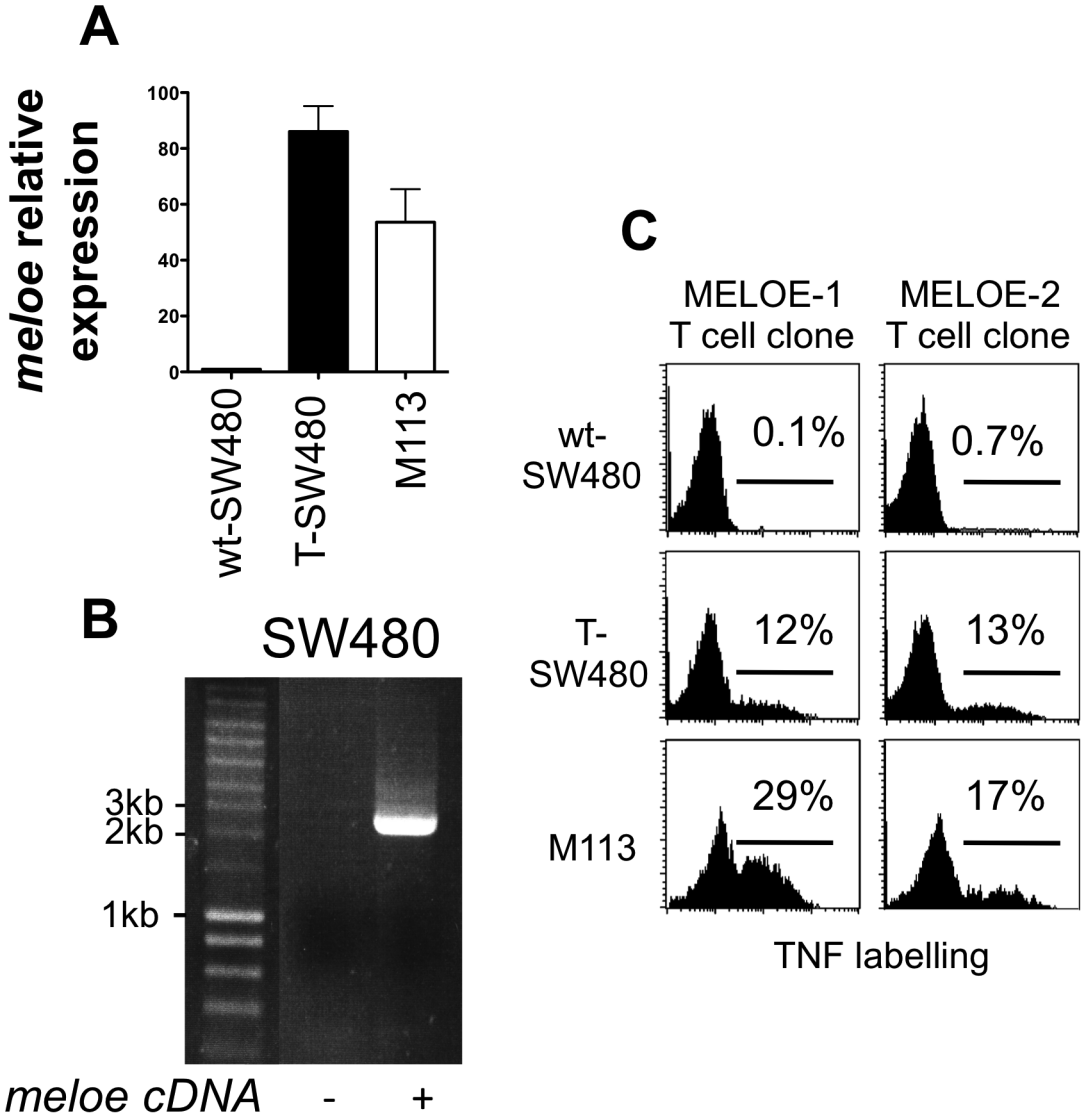
Expression of *meloe* mRNA and MELOE antigens in SW480 line transfected with full-length *meloe* cDNA. A. *Meloe* relative expression measured by RT-qPCR using 3′DR primers on SW480 cells wild type (wt-SW480) or transfected (T-SW480) with full-length *meloe* cDNA. M113 cells were used as positive control. B. RT-PCR products amplified from RNA of SW480 cells transfected or not with full-length *meloe* cDNA. Reverse transcriptions were performed on total RNA samples followed by PCR using 5′end and 3′end primers described on Figure 1C. C. TNF secretion by MELOE-1 (M170.48) and MELOE-2 (M170.51) T cell clones in response to SW480 cell line. Co-cultures were performed at 1∶2 clone:SW480 cells ratio and responses were assessed by TNF-α intracellular staining.

To buttress the hypothesis that both MELOE-1 and MELOE-2 can be translated from the full-length *meloe* transcript, we transfected the SW480 colon carcinoma cell line with the full-length *meloe* cDNA and tested its recognition by MELOE-1/A2 and MELOE-2/A2 specific T cell clones. As previously described [10], SW480 cells expressed very low levels of the *meloe* transcript. After transfection, the level of *meloe* mRNA expression in SW480 cells, estimated by quantitative PCR using 3′DR primers (figure 1C), was greater than that of the M113 melanoma cell line (figure 2A). Again, in both untransfected and transfected SW480 cells, no other transcript than the full length *meloe* could be detected (figure 2B).

As shown on figure 2C, untransfected SW480 cells were not stimulatory for neither MELOE-1 nor MELOE-2 specific T cell clones. In contrast, SW480 cells transfected with *meloe* cDNA were recognized by both T cell clones (figure 2C). This further supported that both MELOE-1 and MELOE-2 antigens can be translated from *meloe* mRNA in transfected SW480 cells.

### Absence of Cryptic Promoter Activity Upstream of MELOE-1 and MELOE-2 ORFs

**Figure 3:**
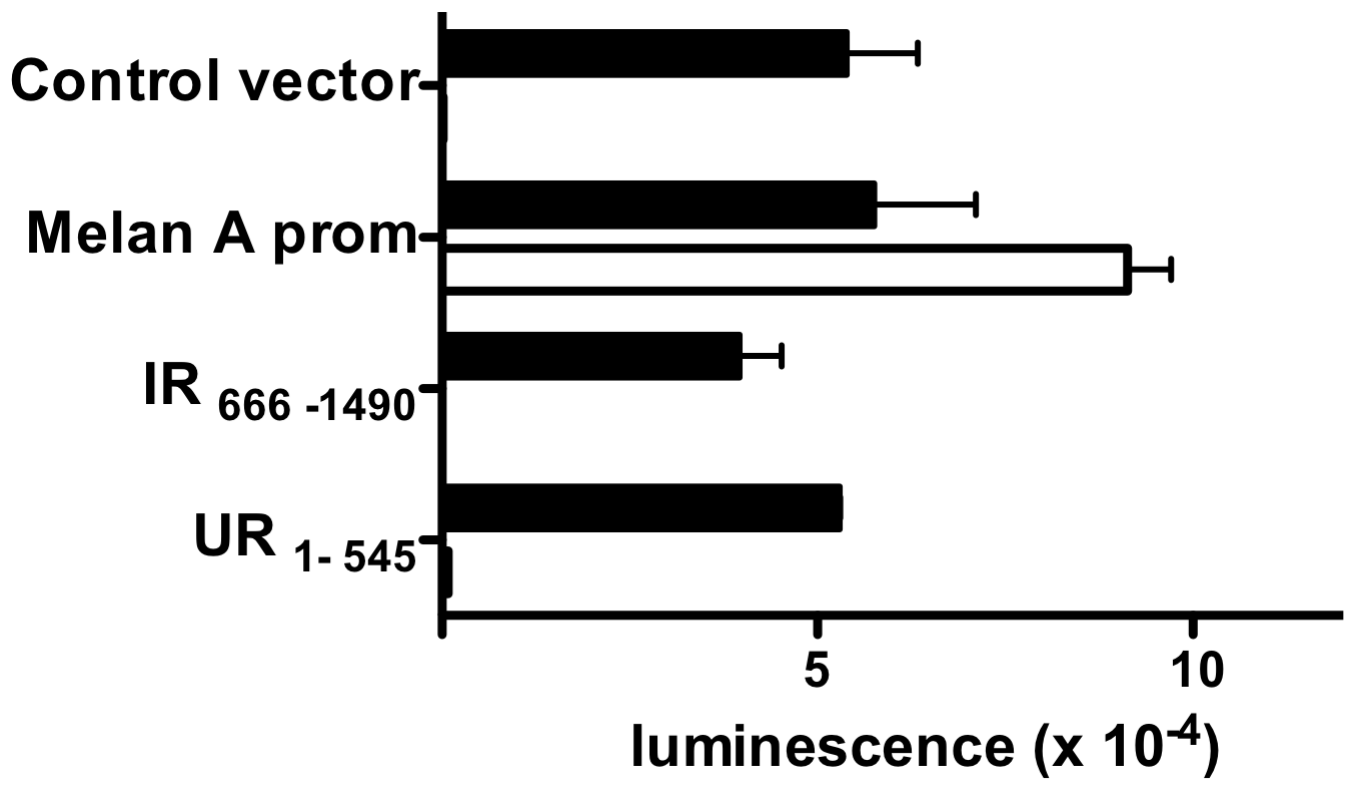
Detection of putative cryptic promoter activity in intercistronic region (IR) and upstream region (UR) of *meloe*. The SV40 promoter region of PGL4-13 vector was replaced by fragments of interest, i.e intercistronic region (IR_666–1490_) between MELOE-1 and MELOE-2, MELOE-2 Upstream Region (UR_1–545_), or the 600 bp melanA promoter as positive control. PGL4-13 without the SV40 promoter was used as negative control. A Renilla luciferase expression plasmid was co-transfected as a positive control of transfection. Renilla luciferase (black bars) and Firefly luciferase (white bars) activities were measured after 48 h and expressed as arbitrary units. Data are expressed as mean +/− SEM from four distinct experiments.

Despite the absence of detectable splicing events, the possibility remained that cryptic promoter sequences may exist upstream of ORF_546–665_ or ORF_1491–1631_ generating shorter transcripts in melanoma cells that would not be detected by 5′end-3′end PCR. To investigate this hypothesis, we cloned the regions upstream of each ORF in front of the reporter Firefly luciferase cDNA and transfected them into M113 melanoma cell line. We used the previously described Melan-A promoter region, exclusively active in melanoma cells [20] as a positive control. As shown in figure 3, no promoter activity could be detected neither upstream of ORF_546–665_ nor upstream of ORF_1491–1631_. Thus shorter *meloe* transcripts could not be generated by cryptic promoter activities.

### Silencing of *meloe* Expression with a siRNA Located Upstream of the MELOE-2 ORF

Finally, we transfected the M88 melanoma cell line with a siRNA hybridizing upstream of ORF_546–665_ (figure 1C). We figured that if the two ORFs were always associated in melanoma cells, then this siRNA should silence the expression of both MELOE-2 and MELOE-1. Alternatively, if shorter transcripts bearing only ORF_1491–1631_ are present in melanoma cells then they should escape this siRNA silencing. We used the M88 melanoma cell line instead of the previously used M113 cell line because *meloe* mRNA levels were somewhat lower in M88 cells thus optimizing the efficiency of RNA silencing.

**Figure 4:**
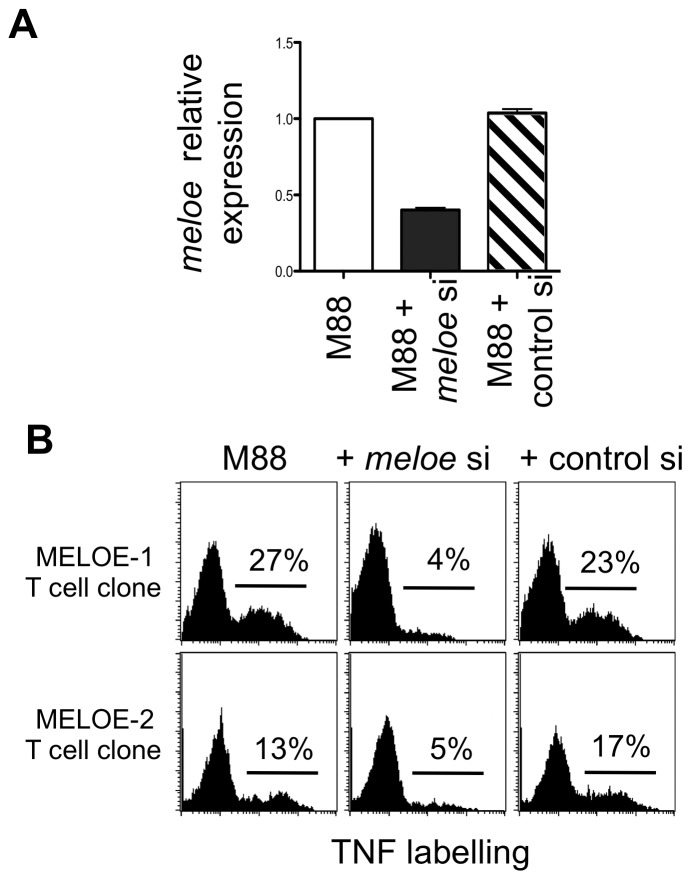
*Meloe* silencing in melanoma cells with a short interfering RNA (siRNA) localized upstream of MELOE-2 ORF. A. M88 melanoma cells were transfected with 50(control si) or siRNA hybridizing 55 bp upstream of MELOE-2 ORF (*meloe* si) along with a GFP reporter plasmid. Relative expression levels of *meloe* mRNA were evaluated on GFP+ sorted cells by qPCR with MELOE-1 primers. B. TNF secretion by MELOE-1 (M170.48) and MELOE-2 (M170.51) T cell clones in response to GFP+ sorted M88 cells transfected with the different siRNAs. Co-cultures with specific clones were performed at 1∶2 clone to melanoma cell ratio and response of each clone was assessed by TNF-α intracellular staining.

Because the efficiency of transfection of M88 cells was only around 50%, we selected transfected M88 cells by co-transfecting a reporter GFP plasmid along with the siRNAs (see m&m) and then sorting GFP+ cells by flow cytometry. The efficiency of *meloe*-specific siRNA silencing was evaluated on sorted GFP+ M88 cells by quantitative PCR using primers located either in the MELOE-1 ORF (i.e. closer to the siRNA hybridization site) or in the 3′region. As shown on figure 4A, using MELOE-1 primers, the specific siRNA (at 50 nM) significantly decreased the expression of *meloe* mRNA in transfected cells while the control siRNA had no effect. Identical results were obtained with qPCR using primers in the 3′region (not shown).

In parallel, GFP+ M88 cells were used to stimulate our two specific T cell clones. Transfection with the *meloe*-specific siRNA at 50 nM dramatically decreased recognition of M88 cells by both MELOE-2 and MELOE-1 specific T cell clones while the control siRNA had no effect on T cell recognition (figure 4B). Thus, silencing MELOE-2 expression with this siRNA resulted in concomitant silencing of MELOE-1 expression. These data further supported the hypothesis that MELOE-1 and MELOE-2 were translated from the same mRNA.

### Evidence for IRES Activities Upstream of MELOE-2 and MELOE-1 Coding Sequences

**Figure 5:**
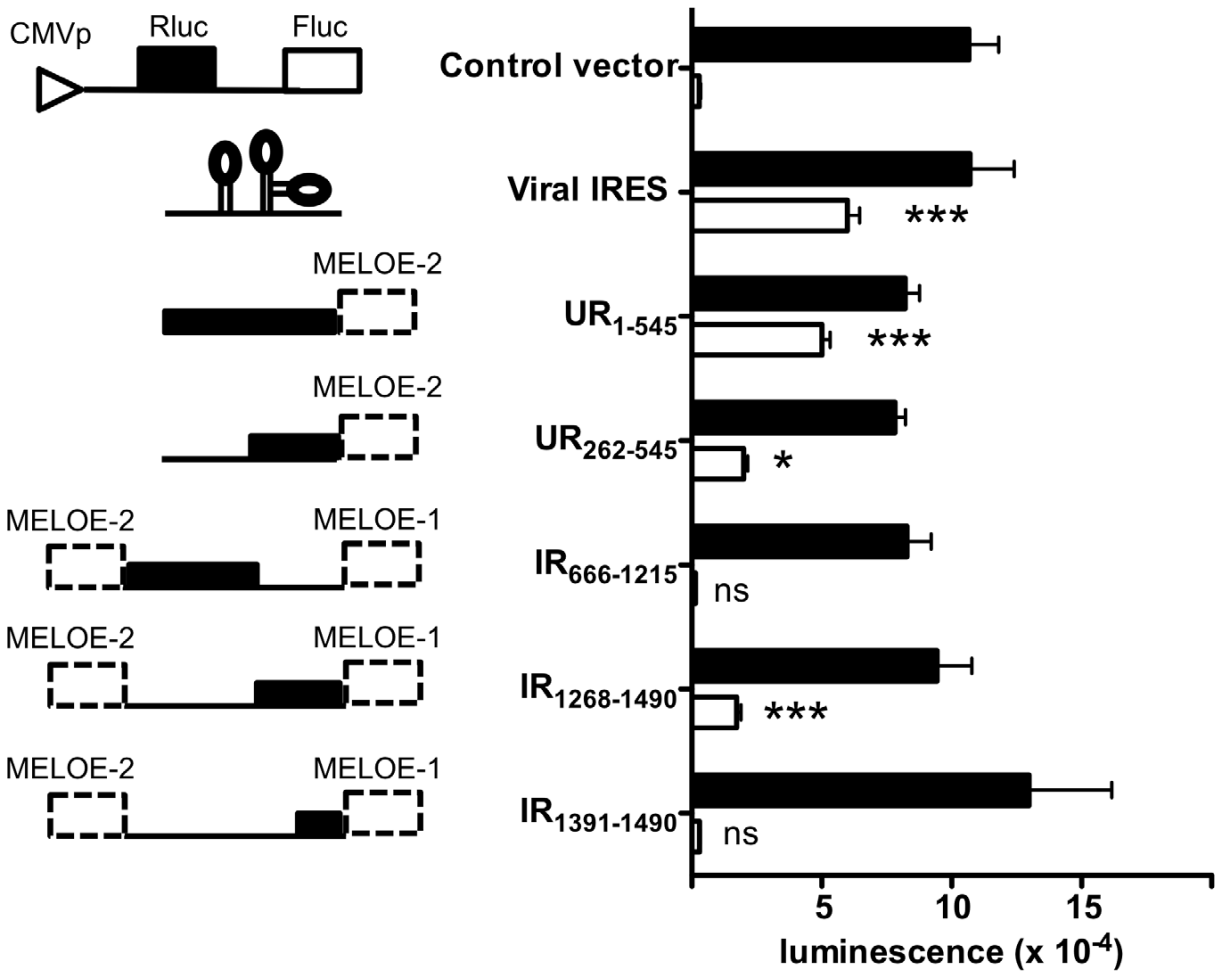
Analysis of IRES activity in upstream region and within the intercistronic region of *meloe* mRNA. M113 melanoma cells were transfected with the pRF bicistronic vector either empty (control vector) or containing the viral EMCV IRES (positive control) or various fragments of *meloe* mRNA: UR_1–545_, UR_262–545_ IR_666–1215_, IR_1268–1490_, IR_1391–1490_. Renilla luciferase (black bars) and Firefly luciferase (white bars) activities were determined 48 h after transfection and expressed as arbitrary units. Data are expressed as mean +/− SEM from four distinct experiments. ***p<0.001, *p<0,05, ns (non significant) by ANOVA and Dunnett post-test.

To define the mechanisms governing MELOE-1 and MELOE-2 translation, we looked for IRES activity upstream of their coding sequences. To this end, we used the previously described Renilla/Firefly bicistronic vector, pRF [21]. After transfection into M113 melanoma cells, we measured the luminescence of each luciferase. As expected, transfection of M113 with the control pRF plasmid resulted in Rluc activity with no Fluc activity while introduction of the well-characterized viral EMCV IRES sequence [22] allowed efficient translation of Fluc (94106±10339 units *vs* 3030±466 units for EMCV *vs* control respectively, n = 4) (figure 5). Cloning of the 5′region of *meloe* upstream of MELOE-2 coding sequence into pRF resulted in significant luciferase activity (50093±3091 units vs 3030±466 units for UR_1–545_ *vs* control, n = 4, p<0.001). When the shorter region UR_262–545_ was cloned into the pRF vector, the efficiency of Fluc translation was significantly decreased (19983±1546 vs 50093±3091 units for UR_262–545_ *vs* UR_1–545_, n = 4, p<0.001) although still significantly higher than the control vector (figure 5). These data strongly suggested the presence of an IRES sequence in front of the coding sequence of MELOE-2.

Similar experiments were performed to explore the intercistronic region between MELOE-2 and MELOE-1 (figure 1C). However, when we subcloned the entire region (IR_666–1490_) into pRF and transfected it into M113 cells, we evidenced a splicing event that took place between a donor site within the Renilla luciferase sequence and an acceptor site located within the IR region (data not shown). Such splicing events have been previously reported with this pRF vector [23]. Thus, we tested shorter fragments covering the entire region. Absence of splicing with these shorter fragments was ascertained by RT-PCR showing a unique amplicon at the expected size (data not shown). The fragment IR_666–1215_ induced no Firefly activity while fragment IR_1268–1490_ did (17285±1668 units for IR_1268–1490_ *vs* 2007±220 units for control, n = 6, p<0.001) (figure 5) thus strongly suggesting the presence of an active IRES sequence in this latter fragment. This IRES activity was abrogated when we shortened the fragment (IR_1391–1490_). Altogether, these results documented the presence of IRES activities upstream of the sequences coding for MELOE-2 and MELOE-1.

### Detection of Proteins Translated from the Full Length *meloe* RNA

**Figure 6:**
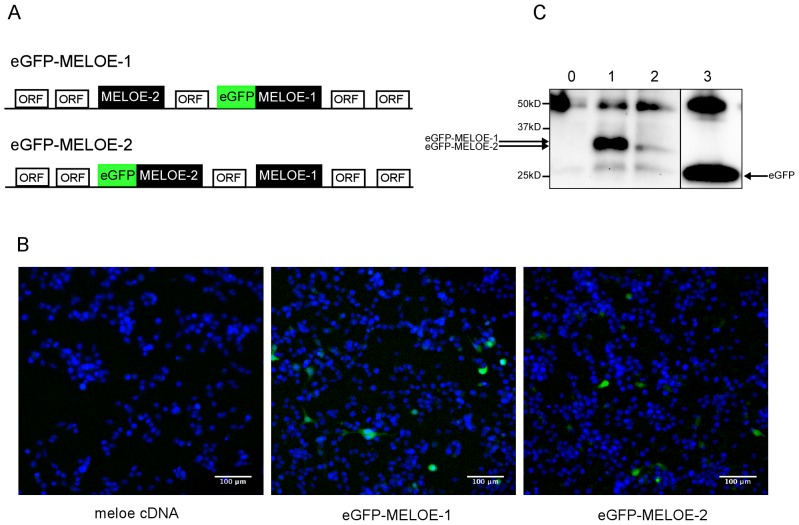
Analysis of MELOE-1 and MELOE-2 expression in melanoma cells with reporter EGFP constructs. A. Schematic representation of eGFP-MELOE-1 and eGFP-MELOE-2 constructs. B. M113 melanoma cells were transfected either with the full length *meloe* transcript (depicted in figure 1C) or with the modified *meloe* constructs shown in A and fluorescence was analyzed 48 h after transfection. Nuclei were stained with Hoescht before analysis. A single representative panel out of the 49 panels analyzed (available as supplemental figures) is shown in each condition. C. Immunoprecipitation with anti-eGFP monoclonal antibody of untransfected M113 cells (lane 0) or transfected with eGFP-MELOE1 (lane 1) or eGFP-MELOE2 (lane 2) or control eGFP vector (lane 3). The same membrane was exposed for 180 s to reveal lanes 0, 1 and 2 and for only 20 s for lane 3. Similar results were obtained in three distinct experiments.

To provide further evidence that the IRES were also functional in the full length *meloe* mRNA, we used eGFP fused to MELOE-1 or MELOE-2 as previously described [16] to allow direct detection of translation in melanoma cells. We designed cDNA contructs in which the sequence coding for fluorescent eGFP was inserted just before MELOE-1 or MELOE-2 ORFs while the rest of *meloe* cDNA was unchanged (figure 6A). We transfected the melanoma M113 cell line with eGFP-MELOE-1 or eGFP-MELOE-2 constructs or with native *meloe* cDNA used as negative control and counted fluorescent cells with an array scan HCS reader. As shown on figure 6B, some melanoma cells became fluorescent after transfection with eGFP-MELOE-1 and eGFP-MELOE-2 but not after transfection with the negative control. The small percentage of fluorescent cells resulted at least in part from a low efficiency of transfection due to the large size of the plasmid constructs (around 8.3 kb) but nevertheless, a clear difference could be seen between control (13/9900 cells with background autofluorescent activity), eGFP-MELOE-1 (473/9600 positive cells) and eGFP-MELOE-2 (144/9500 positive cells) on the 49 image scans performed. (figures S1, S2, S3). Similar results were obtained in two additional experiments. Finally, to confirm expression of eGFP-MELOE-1 and -2 fusion proteins, we performed immunoprecipitations followed by Western blot analyses using a anti-eGFP mAb. A typical experiment is shown in figure 6C: melanoma cells transfected with the eGFP control vector expressed high levels of eGFP protein (27 kD) while melanoma transfected with eGFP-MELOE-1 (lane 1) or eGFP-MELOE-2 (lane 2) expressed the fusion proteins at the expected size i.e. 31.9 kD for eGFP-MELOE-1 and 30.8 kD for eGFP-MELOE-2.

Altogether, these data provided further evidence that ORF_546–665_ and ORF_1491–1631_ can both be translated from *meloe* full length mRNA in melanoma cells.

## Discussion

Although *meloe* mRNA was described in our earlier publications as the source of two melanoma antigens, MELOE-1 and MELOE-2 that could generate a T cell response [10], [19], we did not explore the mechanisms governing its translation.

In the first part of this report, we present several experimental data to rule out the existence of shorter *meloe* transcripts that could account for the translation of ORF_546–665_ (MELOE-2) and ORF_1491–1631_ (MELOE-1) in a classical cap-dependent fashion. Indeed, a report pointed out a number of unjustified claims of non classical translation mechanisms due to undetected levels of splicing or cryptic promoters [24]. We first showed that only the full length *meloe* mRNA could be amplified by a 5′-3′end PCR from either melanoma cell lines, melanocytes or the SW480 cell line transfected with *meloe* cDNA. We also did not detect any cryptic promoter activity upstream of MELOE-2 ORF or upstream of MELOE-1 ORF. Finally, silencing of *meloe* mRNA in the melanoma cell line M88 with a siRNA that hybridized upstream of MELOE-2 ORF led to almost complete abrogation of recognition by both MELOE-2 and MELOE-1 specific T cell clones. We thus concluded that both ORFs were translated from the same mRNA and that *meloe* is at least a bicistronic mRNA. Several examples of polycistronic mRNA have been previously described in insects [16], [25], [26], [27], [28] and in fewer instances in mammals [29], [30], [31], [32]. In these examples, two translation mechanisms have been described to allow expression of downstream ORFs: defective ribosome scanning [27], [33] or internal ribosomal entry sequence (IRES)-dependent translation [32], [34]. Our experiments with bicistronic reporters strongly supported the existence of IRES sequences upstream of both MELOE antigens. For MELOE-2, our data indicated that the full upstream region (UR) was necessary for optimal translation although the shorter UR_262–545_ region still retained significant activity. The precise localization of the putative IRES is further complicated by the existence of two potential ORFs (ORF_132–296_ and ORF_197–370_) in the UR whose translation is currently being investigated. For MELOE-1, IRES activity was present within the 223 bp sequence upstream of MELOE-1 ORF (IR_1268–1490_) and abrogated if the fragment was shortened to a 100 bp sequence (IR _1391–1490_) (figure 5B). We provided additional evidence on the functionality of these IRES sequences within full length *meloe* mRNA by showing that GFP could be translated in melanoma cells when fused with either MELOE-1 or MELOE-2 coding sequences.

Cellular IRES are usually very poorly active *in vitro* because RNA secondary structures are not sufficient to allow recruitment of the 40S ribosomal subunit. They require help from IRES trans-acting factors (ITAF) [35]. We demonstrated that MELOE-1 and MELOE-2 translation occurred in melanoma cell lines and in *meloe*-transfected SW480 since these cells could present epitopes to T cell clones while normal melanocytes were poor stimulators. In addition, we previously showed that these melanocyte cell lines could be recognized by a Melan-A/HLA-A2 specific T cell clone and thus are not defective in antigen processing and presentation [10]. It is thus tempting to speculate that the ITAFs governing this translation may be specifically activated in cancer cells and silent in melanocytes. Indeed, a number of alterations of translation control including IRES activation have been reported in various cancers (for review [36]): for example, the group of Mikulits has shown that the ITAF La was activated during epithelial to mesenchymal transition in hepato-carcinoma allowing IRES-driven translation of laminin B1 [37]. Likewise, in melanoma, hypoxia–induced upregulation of cathepsin L was shown to be regulated post-transcriptionally by an IRES sequence [38].

Our current working hypothesis is thus that the expression of MELOE-1 and MELOE-2 antigens is under a double control, at the transcriptional and translational level. The transcriptional control confers specificity for the melanocyte lineage (Bobinet *et al.*, submitted) whereas the IRES dependent translation confers specificity for melanoma cells. Future characterization of ITAFs involved in the translation of MELOE antigens will be required to confirm this hypothesis. Antigens produced from IRES-dependent translation of the polycistronic *meloe* mRNA (MELOE-1, -2 and possibly others) may thus represent a new class of tumor antigens in that they seem to be both tumor-specific and lineage specific and thus ideal targets for immunotherapy of melanoma.

## Supporting Information

Figure S1 **Montage of the 49 fields analysed in each transfection conditions shown in** **figure 6** **.** Melanoma cells transfected with full length *meloe* without eGFP (S1). Fluorescence was analyzed with an automated fluorescence High Content Screening (HCS) microscopic system (Array Scan VTI, ThermoScientific) and Orca ER camera (Hamamastu). Nuclear staining was performed with 20 µM Hoeschst 33342 (Sigma). Overlay fluorescent images of Hoechst-stained nuclei and GFP labelled cells were acquired using 386/420 nm and 485/515 nm excitation/emission filter couplings, with a 10X objective.
(TIF)

Figure S2 **Montage of the 49 fields analysed in each transfection conditions shown in** **figure 6** **.** Melanoma cells transfected with full length *meloe* without eGFP. Fluorescence was analyzed with an automated fluorescence High Content Screening (HCS) microscopic system (Array Scan VTI, ThermoScientific) and Orca ER camera (Hamamastu). Nuclear staining was performed with 20 µM Hoeschst 33342 (Sigma). Overlay fluorescent images of Hoechst-stained nuclei and GFP labelled cells were acquired using 386/420 nm and 485/515 nm excitation/emission filter couplings, with a 10X objective.
(TIF)

Figure S3 **Montage of the 49 fields analysed in each transfection conditions shown in** **figure 6** **.** Melanoma cells transfected with eGFP-MELOE-2 construct. Fluorescence was analyzed with an automated fluorescence High Content Screening (HCS) microscopic system (Array Scan VTI, ThermoScientific) and Orca ER camera (Hamamastu). Nuclear staining was performed with 20 µM Hoeschst 33342 (Sigma). Overlay fluorescent images of Hoechst-stained nuclei and GFP labelled cells were acquired using 386/420 nm and 485/515 nm excitation/emission filter couplings, with a 10× objective.
(TIF)
