# Supplementary figures and images for: The Melanoma Antigens MELOE-1 and MELOE-2 Are Translated from a Bona Fide Polycistronic mRNA Containing Functional IRES Sequences
Source: PLoS One. 2013 Sep 25;8(9):e75233. doi: 10.1371/journal.pone.0075233 (PMC3783476; doi:10.1371/journal.pone.0075233)

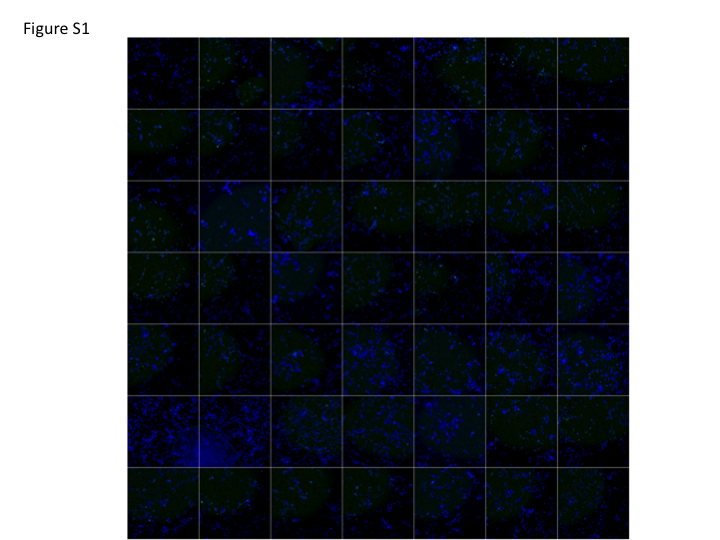

Supplement: Figure S1 — Montage of the 49 fields analysed in each transfection conditions shown in figure 6 . Melanoma cells transfected with full length meloe without eGFP (S1). Fluorescence was analyzed with an automated fluorescence High Content Screening (HCS) microscopic system (Array Scan VTI, ThermoScientific) and Orca ER camera (Hamamastu). Nuclear staining was performed with 20 µM Hoeschst 33342 (Sigma). Overlay fluorescent images of Hoechst-stained nuclei and GFP labelled cells were acquired using 386/420 nm and 485/515 nm excitation/emission filter couplings, with a 10X objective. (TIF) [file pone.0075233.s001.tif]

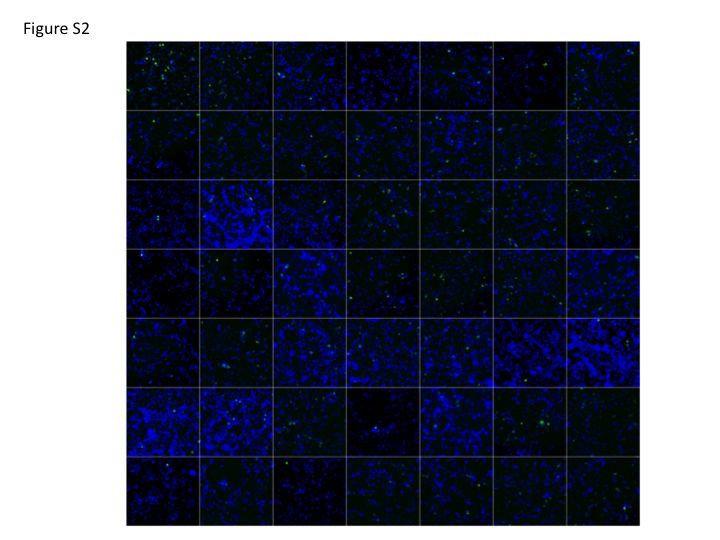

Supplement: Figure S2 — Montage of the 49 fields analysed in each transfection conditions shown in figure 6 . Melanoma cells transfected with full length meloe without eGFP. Fluorescence was analyzed with an automated fluorescence High Content Screening (HCS) microscopic system (Array Scan VTI, ThermoScientific) and Orca ER camera (Hamamastu). Nuclear staining was performed with 20 µM Hoeschst 33342 (Sigma). Overlay fluorescent images of Hoechst-stained nuclei and GFP labelled cells were acquired using 386/420 nm and 485/515 nm excitation/emission filter couplings, with a 10X objective. (TIF) [file pone.0075233.s002.tif]

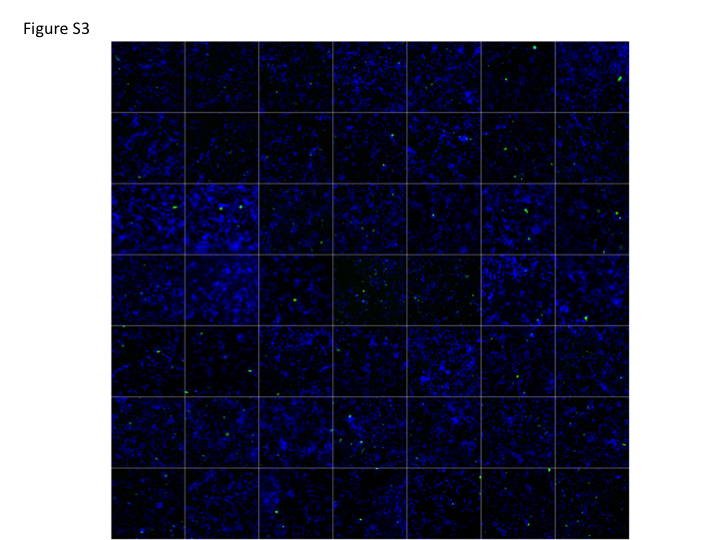

Supplement: Figure S3 — Montage of the 49 fields analysed in each transfection conditions shown in figure 6 . Melanoma cells transfected with eGFP-MELOE-2 construct. Fluorescence was analyzed with an automated fluorescence High Content Screening (HCS) microscopic system (Array Scan VTI, ThermoScientific) and Orca ER camera (Hamamastu). Nuclear staining was performed with 20 µM Hoeschst 33342 (Sigma). Overlay fluorescent images of Hoechst-stained nuclei and GFP labelled cells were acquired using 386/420 nm and 485/515 nm excitation/emission filter couplings, with a 10× objective. (TIF) [file pone.0075233.s003.tif]
